# Risk of Thromboembolism Following Body-Contouring Surgery After Massive Weight Loss

**Published:** 2015-05-22

**Authors:** M. Griffin, M. A. Akhavani, N. Muirhead, A. N. M. Fleming, M. Soldin

**Affiliations:** Plastic and Reconstructive Department, St Georges Hospital NHS Trust, Tooting, London, England

**Keywords:** thromboembolism, deep vein thrombosis, body contouring, massive weight loss, pulmonary embolism

## Abstract

**Background:** “Postbariatric” patients are at significant risk for increased postoperative complications. This study aimed to define the risk of venous thromboembolism following body-contouring surgery after massive weight loss. **Methods:** A retrospective analysis was performed on all patients who had undergone all forms of body-contouring procedures after massive weight loss between January 2005 and August 2012 at St George's Hospital, South West London, United Kingdom. Data were collected on patient demographics, comorbidities, risks factors for thromboembolism, preoperative and postoperative body mass index, and type of surgery. **Results:** A total of 135 operations were performed on 53 patients (43 females, 10 male), with an average age of 44.8 years (range, 26–56 years). Most had staged procedures including 55 abdominoplasties, 23 brachioplasties, 31 thigh lifts, 14 lower-body lifts, and 12 mastopexies. All patients received venous thromboembolism prophylaxis postoperatively including low-molecular-weight heparin (dalteparin) within an average of 22.5 hours after surgery and the application of intraoperative graduated compression stockings. Patients received dalteparin for an average of 4 days (range, 2–14 days), which correlated to their length of stay. One patient had a deep venous thrombosis 14 days postoperatively and then 2 days later developed a nonfatal pulmonary embolus, giving a venous thromboembolism prevalence of 0.74% (1/135). **Conclusions:** The clinically apparent venous thromboembolism prevalence was low among patients undergoing body-contouring procedures after massive weight loss in this study. We provide evidence of a successful algorithm to prevent venous thromboembolism for patients undergoing body-contouring procedures after massive weight loss.

Postbariatric patients present a unique challenge to the plastic surgeon as not only do they have complex reconstructive challenges but also they have residual medical comorbidities, nutritional deficiencies, and psychological problems.^1^ Hence, careful patient selection is essential to minimize significant morbidity and mortality.

The overall incidence of deep venous thrombosis (DVT) in hospitalized patients varies according to the surgery they have undergone, underlying pathology, and illness severity. General medical patients have a risk of 10% to 20%, general surgery patients have a risk of 15% to 40%, and patients after hip fractures have a risk of 40% to 60%.^2^ In comparison, the incidence of venous thromboembolism (VTE) in plastic surgery is reported to be as low as 1% to 2%.^3^^-^6 The aim of this study, was to analyze the risk of VTE following all cases of body-contouring procedures after massive weight loss.

## METHODS

A retrospective analysis of all patients undergoing body-contouring surgery after massive weight loss between January 2004 and August 2012 was performed. The surgery was usually completed in stages. Data were collected by 2 authors independently (M.G. and N.M.), and any discrepancies were resolved by consensus. Data that were collected included patient demographics, patient care pathways, perioperative events and any postoperative complications, length of hospital stay, discharge plans, and the nature and number of further operations. VTE prophylaxis pre- and postoperatively, including chemical and mechanical prophylaxis, was also recorded.

Prior to surgery, patients were stratified according to their VTE risk into low, medium, or high risk, based on patient and surgical factors (Table 1). Different patient and surgical factors are given individual scores and then summed together to gain the overall score. Depending on their risk stratification score, they would receive an appropriate VTE prophylaxis regimen (Table 2). Patients were followed up until the time of final discharge (all being >12 months). If a patient receives a score of 3, and hence is considered to be at high risk for VTE, hematology would be consulted to assess this patient's suitability for surgery.

### Statistical analysis

Statistical analysis was performed using Excel 2007 and Stats Direct statistical analysis software. For univariate analysis, dichotomous data were analyzed using the Fisher exact test. A binary logistic regression analysis was used to establish the effect of continuous data on the number of surgical complications. A *P* value of less than .05 was considered statistically significant.

## RESULTS

One hundred thirty-five procedures were carried out on 53 patients over the 7-year period from January 2005 to August 2012. The cohort included 43 female and 10 males, with an average age of 44 years (range, 23–59 years). Fifty-five abdominoplasties, 31 thigh lifts, 23 brachioplasties, 12 mastopexies, and 14 lower-body lifts were carried out during the study period. Table 3 shows a range of comorbidities and ASA (American Society of Anesthesiologists) scores in the patient group. Weight loss prior to body contouring was due to gastric bypass in 18 patients, gastric banding in 20 patients, and diet and exercise in 15 patients. The mean body mass index (BMI) prior to their weight loss procedure was 44.8 kg/m^2^ (range, 30.9–60.9 kg/m^2^), and mean BMI prior to body contouring was 28.2 kg/m^2^ (range, 24–44 kg/m^2^). This led to a mean excess weight loss of 36% prior to the body-contouring procedure (range, 20%–50%).

Each patient had a mean of 3 body-contouring procedures (range, 1–6), with an overall mean operative time of 3.68 hours (range, 2–7 hours). All patients received intravenous antibiotic prophylaxis at induction. Mean inpatient stay was 4 days (range, 2–14 days). The average total time under the care of plastic surgery was 32 months (SD = 19) and from initial bariatric surgery referral to discharge from plastic surgery was 96 months (SD = 26).

### Complications after body contouring

Major complications were defined as readmission to hospital or return to operating theater. In the 135 procedures, there were 12 major complications (8.8%) (Table 4). Minor complications were considered those that did require surgical intervention or readmission (Table 5). Combining 2 or more body-contouring procedures on the same day or where the patient had a previous body-contouring procedure did not significantly increase the number of complications (Fisher exact test, *P* = .5787 and *P* = .1790, respectively). The overall number of complications did not differ according to BMI (regression, *P* = .13), gender (regression, *P* = .06), age (regression, *P* = .06), or operation time (regression, *P* = .12).

### VTE practice and complications

According to our risk stratification, no procedures were considered low risk, 115 procedures were considered medium risk, and 20 procedures were considered high risk. Two patients had a previous DVT due to hypercoaguable disorders, 1 patient had a current malignancy, 24 patients were still classified as obese (BMI >30 kg/m^2^), 20 patients were taking hormone replacement therapy/contraceptive pill preoperatively, and 1 patient had a hypercoaguable disorder without experiencing previous DVT or pulmonary embolism (PE).

One patient who was classified as being at high risk because of a previous DVT/PE received preoperative heparin infusion. This patient suffered a postoperative hematoma, which required evacuation. Two patients received an inferior vena cava (IVC) filter prior to surgery because of their risk factor stratification for previous DVT/PE and current malignancy. Neither of these patients suffered any postoperative hematomas.

Preoperatively, 25 patients received 2500 units of low-molecular-weight heparin (LMWH) dalteparin on the evening prior to their surgery at 6 pm. Twelve of these patients developed hematoma, and 7 returned to surgery for evacuation. In 2009, VTE prophylaxis practice changed in our hospital, where patients did not receive preoperative dalteparin but received 5000 units of LMWH dalteparin only after their operation as shown in Table 2. From 2009 onward, there were only 5 hematoma cases, none of which required further surgery. There was a significant decrease in hematoma prevalence when dalteparin was used only postoperatively (χ^2^ test, *P* > .001).

The average number of hours to the patient's first dose of dalteparin postoperatively was 22.44 hours (range, 6–33 hours). The hematoma prevalence was not significantly related to the timing of the postoperative dalteparin (*P* = .266). Patients received only dalteparin while they were hospitalized. No patient had any adverse reactions due to dalteparin.

One patient in this cohort suffered a DVT using this regimen. A female patient developed a DVT after abdominoplasty. This patient had a preoperative BMI of 40 kg/m^2^, which posed a risk for DVT. Obesity alone was not considered to be an indication for a preoperative IVC filter. The patient received dalteparin 24 hours postsurgery. The patient wore graduated compression stockings (GCS) pre- and postoperatively. The patient developed unilateral leg swelling 14 days postsurgery while at home and was admitted into a nearby hospital where a scan confirmed a DVT. A further 2 days later, the patient developed a nonfatal PE. The patient was started on warfarin for 6 months.

## DISCUSSION

The purpose of this study was to assess the risk of DVT and PE following body-contouring surgery after massive weight loss. With the increase in the number of body-contouring procedures, it is important for surgeons to be able to predict whether patients are at a greater risk of postoperative complications, so they can select the most appropriate treatment option.

A recent survey by the American Society of Plastic Surgeons revealed that for postbariatric body-contouring surgery, DVT has occurred in more than one third of plastic surgeons’ practices, with 7% of surgeons reporting a patient death from PE, and the authors concluded that more studies are required in this field to improve patient care and safety.^7^ There is a lack of clear guidance for the prevention of VTE in plastic surgery. Grazer and Goldwyn^4^ reported an incidence of 1.2% for DVT and 0.8% for PE in a series of abdominoplasties. Other studies have shown that the incidence of VTE rises when abdominoplasties are combined with another intra-abdominal procedure, with incidence rising up to 6.6%.^8^ It seems reasonable to assume that VTE complications would be higher in patients undergoing body-contouring surgery for the following reasons: (1) the length of procedures; (2) incidence of PE, which rises to 3.5% in obese and bariatric patients even when using heparin prophylaxis; and (3) decreased venous return from the lower extremity due to intraoperative positioning.^9^

Understanding the effect of obesity on the risk of postoperative surgical complications is of great importance to the surgical field. Harth et al^10^ observed obese patients who underwent hernia repair and found an increased wound complication rate after concurrent ventral hernia repair and panniculectomy, with an odds ratio of 5.4 (*P* = .04). Fischer et al^11^ similarly found that obesity was a risk factor for major operative morbidity after abdominal wall reconstruction in 1081 patients. Similarly, Fisher et al^12^ observed that ventral hernia repair and panniculectomy in states of extreme obesity (BMI ≥45.0 kg/m^2^) is associated with a higher incidence of complications and as such staged procedures should be given consideration. Systemic surgical complications including DVT/PE have also been reviewed in obese patients. Nelson et al^13^ reviewed the 2005–2010 American College of Surgeons National Surgical Quality Improvement Program database and identified the cases of abdominal wall reconstruction and examined early complications in the context of obesity. Pulmonary embolism incidence was nearly 10 times higher in obese patients (2.1% vs 0.2%; *P* = .001) and as such VTE overall was additionally significantly higher (2.8% vs 0.8%; *P* = .006).^13^

The risk of surgical complications after body-contouring surgery for the bariatric versus nonbariatric patients has been reported to be higher. Greco et al performed a large retrospective study in 222 patients and observed a greater risk of wound surgical complications after abdominoplasty and panniculectomy in bariatric patients (*n* = 139) than in nonbariatric patients (*n* = 83) (41% vs 22%; *P* < 0.01).^14^ Similarly, early complications were significantly higher in postbariatric patients (48%) than in patients who had not had weight loss surgery (29%) in 161 patients undergoing abdominoplasty.^15^ However, a prospective study illustrated no evidence for an association between the weight loss method and risk in patients undergoing body-contouring surgery in 34 procedure-matched case controls.^16^ Further studies that examined the risk of DVT/VTE, specifically after body contouring, have also been carried out. Reish et al^17^ found no clinically detected DVT in 105 patients of whom 62% had massive weight loss. In this study, high-risk patients received 5000 units of low-dose unfractionated heparin (LDUH) preoperatively and 5000 units of LDUH 8 to 24 hours postoperatively with the addition of pneumatic compression devices.^17^ In a cohort of 3334 patients, of whom 229 patients had massive weight loss, Pannucci et al^18^ illustrated a risk reduction in high-risk patients on using enoxaparin 6 to 8 hours postoperatively. Including this study, the literature to date illustrates a low detection rate of VTE in patients undergoing body-contouring surgery after massive weight loss. Current studies have also documented that the rate of VTE after bariatric surgery is less than 1%, which is similar to the low VTE risk after body contouring.^19^ In our series, we have shown a prevalence of VTE of 1.89% after body-contouring procedures. A recent meta-analysis by Hasanbegovic and Sørensen^20^ reviewed the postoperative complications after body-contouring surgery after weight loss. Seven studies were found to be eligible to be included in the study. The meta-analysis revealed that 60% to 87% patients had increased risk of having a postoperative complication, having previously undergone weight loss surgery compared with weight loss after dietary changes or exercise.^20^ The authors concluded that local complications, such as hematoma, infection, seroma, and dehiscence, were high, whereas systemic complications, such as DVT or PE, were rare, with only 1 study showing evidence of DVT in postbariatric patients (2/23).^20^

The correct type, dosage, and timing of chemoprophylaxis for the surgical patient population remain a controversial topic in both the literature and ordinary daily practice. LMWH has been found to be an effective chemoprophylaxis in general surgery and orthopedic surgery patients and recently for plastic surgery.^21^ Seruya et al^22^ found that pneumatic compression plus subcutaneous heparin conferred a statistically significant reduction in the rate of VTE without a significant increase in bleeding versus mechanical prophylaxis alone after 173 operations, which involved 120 patients at high risk for VTE. Furthermore, Pannucci et al^18^ found that in high-risk plastic surgery patients, postoperative enoxaparin prophylaxis is protective against 60-day VTE when controlling for baseline risk and length of stay. Hatef et al^1^ illustrated that enoxaparin confers a significant advantage in preventing VTE in high-risk patients. Compared with LDUH, LMWH is easier to titrate because of its selective binding to factors XA and IIA, without binding other plasma proteins. Other advantages of LMWH include that it requires only once-daily dosing and has better bioavailability as a subcutaneous injection. Furthermore, LMWH has a long half-life and therefore has less chance of developing heparin-induced thrombocytopenia.^9^ There have been reports that LMWH has a dose-related effect on bleeding complications.^23^ However, reports have shown that as long as dosing is below 3400 units, LMWH is as effective as LDUH in preventing VTE and has low bleeding complications.^2^ With no common standard on reporting postoperative complications and VTE, it is difficult to compare different treatment regimens. Selection of an optimal VTE prophylaxis pathway may be discovered if there were common standards.

Appropriate timing of administration of the first dose of chemoprophylaxis is not clear. Studies have shown that administration of the first dose of chemoprophylaxis within 6 hours can significantly increase the risk of bleeding.^24^ We found a significant difference in hematoma formation in patients receiving preoperative dalteparin, which led to a change of our practice of giving dalteparin only postoperatively. The average timing of the first dose after surgery varied because of daily ward practicalities, but still we achieved a low VTE prophylaxis prevalence overall.

Our hospital policy states that IVC filters should be used in patients with significant risk factors for DVT in preference to the use of intravenous preoperative heparin administration due to the significant risk of bleeding associated with this practice, which we observed in our early cohort. The single case of DVT in our cohort may have been prevented by the use of a preoperative IVC filter. However, there are risks with the insertion of these devices, which should be considered.^25^^,^^26^ Several studies have reviewed the use of IVC filters in high-risk patients. Unfortunately, further prospective studies are required to examine individuals at high risk of DVT to fully understand which exact patients would benefit from IVC filters.

All of our patients wore GCS, as it is well documented in the literature that these decrease the incidence of DVT by passively applying constant pressure, which reduces the volume of blood that remains in the leg, decreasing vein distension and stasis.^27^ Some reports have shown that they reduce plasminogen activator 1 levels and the release of tissue plasminogen activator.^28^ Graduated compression stockings are highly effective, with reports showing a 60% reduction in DVT risk.^27^ We apply GCS prior to surgery, which has shown to be beneficial because patients are most susceptible to venodilatation and venous stasis at induction and during surgery.^20^ We also encourage patient mobilization from day 1 after surgery.

We propose that using risk stratification algorithms is a useful and effective method to prevent VTE. A particularly difficult scenario is the treatment of very high-risk patients taking warfarin because of previous DVTs or PEs. For these patients, our hospital policy states that warfarin be stopped and an IVC filter be fitted 1 week before surgery. Our standard postoperative regimen of 5000 units of subcutaneous dalteparin is then prescribed with removal of the filter a week later when the patient is mobile. When VTE prophylaxis is being considered prior to surgery, clinicians should follow the local hospital guidelines in conjunction and agreement with the surgical team's experience and evidence-based medicine.^29^ The American College of Chest Physicians^2^ published guidelines on the prevention of VTE for various surgical specialties, but it was not specifically applicable for plastic surgery patients. Therefore, Davison et al^30^ in 2004 modified the Caprini model to make the VTE prophylaxis more suitable to plastic surgery patients.

This study was retrospective and therefore the rate of silent or nonclinical DVT remains unknown. A prospective study using ultrasonography at multiple postoperative time intervals to find the true incidence of VTE following body-contouring procedures after massive weight loss is needed.

## CONCLUSION

It is clear that VTE remains controversial in surgery, with various governing bodies publishing recommendations for this preventable postoperative complication. With numerous studies highlighting that patients with massive weight loss are at greater risk of postoperative complications, documenting VTE risk following body-contouring surgery after massive weight loss is important. There is a need for larger prospective studies with appropriate controls to fully understand the risk of VTE following body-contouring surgery after massive weight loss.

## Figures and Tables

**Table 1:** The risk factor stratification tool used to work out postoperative risk for VTE for surgical patients*

Patient thrombosis risk factors	Factor	Admission-related thrombosis risk factors	Factor
Age 40–60 y	1	Significantly reduced mobility >3 d	1
Age >60 y	2	Hip/knee replacement	2
History of VTE	3	Surgical procedure + total anesthetic time >90 min	2
Current pregnancy	1	Surgical procedure involving pelvis or lower limb and total anesthetic + procedure time >60 min	2
Severe sepsis/infection	1	Critical care admission	1
Acute of chronic lung disease	1	Anticipated bed rest >4 d	1
Cardiac failure/recent MI	1	Immobilizing plaster cast	1
Dehydration	1	Lower limb paralysis (excluding acute stroke)	1
Current malignancy	2		
Obesity (BMI >30 kg/m^2^)	1		
OCP/HRT	1		
Hypercoagulable disorder	3		
Nephrotic syndrome (albumin <30)	1		
Myeloproliferative disease	1		

*VTE indicates venous thromboembolism; MI, myocardial infarction; BMI, body mass index; and OCP/HRT, oral contraceptive pill/hormone replacement therapy.

**Table 2:** Venous thromboembolism interventions used for body contouring surgical patients based on risk factor profile*

Number of factors	Risk factor	Intervention
1	Low risk	*Preoperative*: Consider preoperative GCS for low-risk surgical patients
		*Intraoperative*: Consider GCS and PCD if operation >4 h
		*Postoperative*: No intervention, consider GCS for low-risk surgical patients
2	Moderate risk	*Preoperative*: GCS
		*Intraoperative*: GCS and PCD if operation >4 h
		*Postoperative*: 5000 units of dalteparin SC once daily and GCS
3	High risk	*Preoperative*: GCS and speak to hematology at the preoperative clinic because an IVC filter may be needed
		*Intraoperative*: GCS and PCD if operation >4 h.
		*Postoperative*: 5000 units of dalteparin SC once daily and GCS

*GCS indicates graduated compression stockings; PCD, pneumatic compression devices; IVC, inferior vena cava; SC, subcutaneously.

**Table 3:** Comorbidities and ASA scores of patients undergoing body-contouring surgery^*^

Comorbidities	ASA score
HTN: 13	1:21
Diabetes: 5	2:31
Depression: 17	3:1
OA: 4	
Angina: 6	
Smoking status: 27	
HIV infection: 1	
Epilepsy: 2	
GORD: 12	
Cancer (porocarcinoma): 1	
Obstructive sleep apnea: 8	

*ASA indicates American Society of Anesthesiologists; HTN, hypertension; OA, osteoarthritis; and GORD, gastroesophageal reflux disease.

**Table 4:** List of major complications within the cohort*

Body-contouring procedure	Reason for return to operating theatre	Time to return to operating theatre, d
Abdominoplasty	Evacuation of hematoma and washout	2
Upper-body lift and mastopexy	Evacuation of hematoma	3
Lower thigh lift and brachioplasty	Evacuation of hematoma	1
Abdominoplasty and thigh lift	Evacuation of hematoma	1
Revision of abdominoplasty	Evacuation of hematoma	2
Lower thigh lift	Evacuation of hematoma	2
Brachioplasty	Evacuation of hematoma	4
Abdominoplasty, mastopexy, and brachioplasty	Wound debridement—application of VAC dressing	14
Revision of abdominoplasty	Wound dehiscence—washout	7
Abdominoplasty	Wound dehiscence—debridement and application of skin graft to abdomen	90
Lower-body lift	Incision and drainage of seroma	14
Abdominoplasty	Surgery for surgical site infection	3
Total		12

*VAC indicates vacuum assisted closure.

**Table 5:** List of minor complications within the cohort

Minor complication	*n*
Seroma	4
Hematoma	10
Wound dehiscence	10
Surgical site infection	12
Lymphedema	1
Total	37
